# H3K14: A histone site closely related to diseases

**DOI:** 10.7150/jca.118273

**Published:** 2025-07-28

**Authors:** Chenyu Fan, Feng Zeng, Yan Lei, Qian He, Kun Liu, Juan Xu, Yanhong Zhou

**Affiliations:** 1 Department of critical care medicine, The Affiliated Cancer Hospital of Xiangya School of Medicine Central South University/Hunan Cancer Hospital, Changsha, Hunan 410013, China.; 2 Cancer Research Institute, Basic School of Medicine, Central South University, Changsha, Hunan 410011, China.; 3 Department of Blood Transfusion, The Affiliated Cancer Hospital of Xiangya School of Medicine Central South University/Hunan Cancer Hospital, Changsha, Hunan 410013, China.; 4 Department of Radiation Oncology, The Affiliated Cancer Hospital of Xiangya School of Medicine Central South University/Hunan Cancer Hospital, Changsha, Hunan 410013, China.; 5 Department of Neurosurgery, The Second People's Hospital of Hunan Province (Brain Hospital of Hunan Province), Clinical Medical School, Hunan University of Chinese Medicine, Changsha Hunan, 410007, China.

**Keywords:** H3K14, acetylation modification, methylation modification, transcription, DNA repair

## Abstract

Histone H3K14 is a crucial site for histone H3 modifications, which are intimately connected to processes such as DNA replication, gene expression, and transcription. Modifications at H3K14 can lead to damage within the human body. Specifically, the acetylation of H3K14 influences immune system function, often linking to conditions like tumor inflammation and immune-related diseases. Lately, H3K14 has garnered significant attention across various scientific disciplines. This review outlines how H3K14 acetylation impacts DNA replication, enhances gene expression, and influences T cell development and activation. Furthermore, the combination of H3K14 acetylation with propionylation and butyration can also stimulate gene expression. In contrast, H3K14 methylation hinders DNA replication, while H3K14 ubiquitination affects both gene expression and transcriptional activity. The review also discusses the regulation of H3K14 by non-coding RNA, histone acetyltransferases, histone deacetylases, nuclear proteins, and pharmaceutical compounds. It explores the relationship between H3K14 and the diagnosis, onset, and treatment of diseases. By doing so, this review aims to offer a fresh perspective for a comprehensive understanding of H3K14's functions.

## 1. Introduction

In eukaryotes, DNA is packaged into a complex of nuclear proteins known as chromatin. The fundamental repeating unit of chromatin is the nucleosome core particle, which comprises approximately 146 base pairs of DNA wrapped around a core of histones H2A, H2B, H3, and H4, with two molecules of each. H3K14 is a specific modification site on histone H3, formed when the 14th position of the histone is occupied by a lysine residue 1. The core particles of nucleosomes can be organized into a hierarchical structure, creating an environment that restricts access to the DNA template, thereby inhibiting processes such as transcription. Post-translational modifications of histones are a key mechanism for modulating chromatin structure to alter DNA accessibility 2. These modifications, following the addition of acetyl groups, include acetylation, methylation, phosphorylation, ubiquitination, and SUMOylation. Core histones are predominantly globular and can also be covalently modified through unstructured N-terminal or C-terminal tails and globular domains 3. Among the histones, H3 is the most frequently modified, with H3K14 being a common site for both methylation and acetylation. The state of H3K14 modification can influence the development of various diseases, particularly in the context of tumors and immune disorders. In summary, research on H3K14 modification has begun to elucidate its effects and the factors that influence it. However, a comprehensive and systematic overview of the impact of H3K14 modification on human health and disease has yet to be provided.

This article provides a comprehensive review of the functional impacts of H3K14 epigenetic modifications, the various factors that influence these modifications, and the latest research advancements regarding the connection between aberrant H3K14 modifications and immune diseases, as well as potential therapeutic strategies.

## 2. The effect of H3K14 Acetylation on Its Function

Acetylation of H3K14 is a crucial histone modification, often associated with transcriptional activation. This process leads to a cascade of gene expression events. Additionally, the acetylation of H3K14 plays a significant role in modulating immune responses.

### 2.1 Acetylation of H3K14 affects DNA replication

Acetylation of H3K14 (H3K14ac) is a significant modification that promotes DNA replication. The histone acetylation enzyme Sir2 is necessary for telomerase activity. However, it exhibits redundancy with the histone acetylation enzyme Clr3 to maintain centromeric heterochromatin. Sir2 acetylates H3K14 to target Clr4 to the centromere 4. When H3K14 is mutated, it can block the recruitment of Clr4 to the centromere, thereby affecting DNA replication. Additionally, the absence of TBP and H3K14ac in condensed chromatin, along with rDNA retrotransposon enrichment in regions R1 and R2, may lead to epigenetic silencing flanking the rDNA gene 5. A specific mutation in H3K14 can disrupt rDNA silencing. A single mutation in H3K14, including mutations at the N-terminal end of H3, can delay copy-dependent nucleosome assembly and extend the lifespan of the copy. However, H3K14ac inhibits DNA synthesis in DNA repair (base excision repair, BER). During single nucleotide clearance, nucleosome core particles (NCPs) with H3K14ac near the center of the DNA polymerase beta gap-filling activity show strong inhibition, suggesting that acetylation of H3K14 in nucleosomes may promote an alternative gap-filling pathway by inhibiting the activity of DNA polymerase β 6. However, this does not directly indicate the inhibitory effect of H3K14ac on DNA replication, but rather H3K14Ac may indirectly maintain genome stability during replication by inhibiting Polβ (a low-fidelity polymerase) and directing DNA damage to high-fidelity repair pathways (such as long fragment BER or recombination repair). H3K14ac also enhances the chromatin remodeling ability. Nucleosomes with H3K14ac have a higher affinity for the chromatin remodeling complex RSC, which can reshape chromatin structure. This is due to the specific binding of RSC's Sth1BD to H3K14ac 7. H3K14ac compresses and recruits chromatin remodeling through direct regulation of chromatin protein complexes like RSC, enhancing the RSC's activity in nucleosome remodeling and increasing the chromatin remodeling complex's reconstitution activity 8. This indicates that H3K14 acetylation plays a significant role in activating the DNA damage checkpoint.

### 2.2 H3K14 acetylation promotes gene expression

High acetylation levels at H3K14 can influence gene expression, DNA repair, and transcriptional activation. Blocking the acetylation process at H3K14 can lead to the activation of abnormal signaling pathways and gene expression patterns. Under the influence of histone deacetylase inhibitors (HDACi), increased H3K14 acetylation can enhance the expression of the BDNF II promoter, providing essential nutrients to the brain and potentially protecting against ischemic and anoxic injuries 9. Furthermore, the combined action of HDACi and a cAMP agonist can induce glioma stem cells to differentiate into neuron-like cells by regulating H3K14 acetylation 10. Conversely, hypoacetylation of H3K14, caused by the downregulation of acetyltransferases, can inhibit the activity and proliferation of B-ALL cells, leading to an extended cell cycle and increased cell apoptosis. Additionally, H3K14 acetylation can raise the expression of CTNNB1 and activate the Wnt/β-Catenin signaling pathway, which regulates cell differentiation 11. Knockout of HDAC-targeted genes that are targeted by HDAC acetylation enzymes can lead to high acetylation levels at H3K14, inducing the promoters of the cancer-associated gene c-Myc and the miR-a-5p30 gene. The combined effect of promoting miR-a-5p transcription and inhibiting c-Myc gene expression can have significant impacts 12. Low expression of H3K14 acetylation is associated with an increased risk of diseases. Interestingly, Gcn5, a histone acetyltransferase, works in concert with Clr3, a histone deacetylase, to regulate H3K14 acetylation levels and transcriptional elongation. This interaction affects the efficiency of transcriptional elongation of critical stress-response genes 13. These findings on gene expression regulation may offer new therapeutic strategies for cancer treatment.

**Figure 1:**
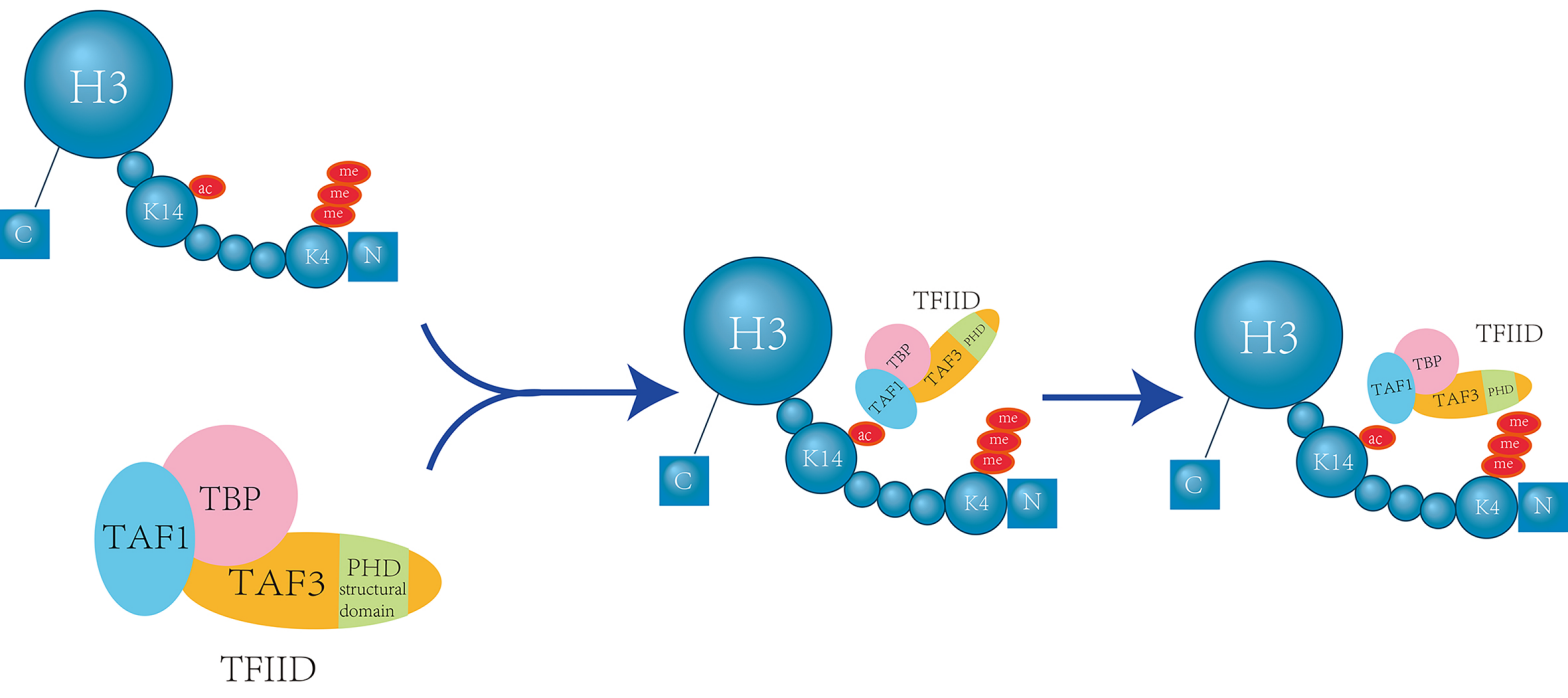
** H3K14 Acetylation Enhances TFIID's Recognition of H3K4me3.** The TAF1 protein, a component of the TFIID complex, can specifically recognize H3K14 acetylation (H3K14ac). This recognition event induces a conformational change in the TAF3 subunit, thereby strengthening the interaction between TAF3's plant homeodomain (PHD) finger and the H3K4me3 mark. This enhancement of binding affinity facilitates the transcriptional function of TFIID. When H3K14 is acetylated, it promotes the progression of this recognition process, thereby stimulating transcriptional activation 14.

In addition to their individual roles, there is a complex interplay between H3K14 acetylation and H3K4 methylation. The basal transcription factor TFIID directly interacts with the H3K4me3 mark through the plant homeodomain (PHD) finger of its subunit TAF3. Importantly, acetylation at H3K14 further enhances this interaction 14 (Figure 1).

This crosstalk between the two modifications can also lead to synergistic effects. Specifically, the double histone modification tags H3K4me1 H3K14ac and H3K4me0 H3K14ac are recognized by the protein ZMYND8 (also known as RACK7) through its plant homeodomain (PHD) and bromodomain. These modifications can play a role in inhibiting gene expression 15. Understanding these mechanisms provides valuable insights that could inform the development of therapeutic strategies for certain diseases.

### 2.3 Acetylation of H3K14 affects T cell development and activation

H3K14 acetylation (H3K14ac) plays a significant role in the human immune system, particularly by influencing T cells. Lysophosphatidylcholine (LPC) has been shown to upregulate trained immunity pathways (TIPs) in human aortic endothelial cells (HAECs) by increasing H3K14 acetylation 16. However, interleukin-35 (IL-35) counteracts this effect by inhibiting H3K14 acetylation. IL-35 inhibits lysophosphatidylcholine (LPC) -induced endothelial cell activation by inhibiting mitochondrial reactive oxygen species (mtROS) and histone H3K14 acetylation (H3K14ac), down-regulating adhesion molecules (such as ICAM-1) expression, and reducing monocyte adhesion 17.

**Figure 2:**
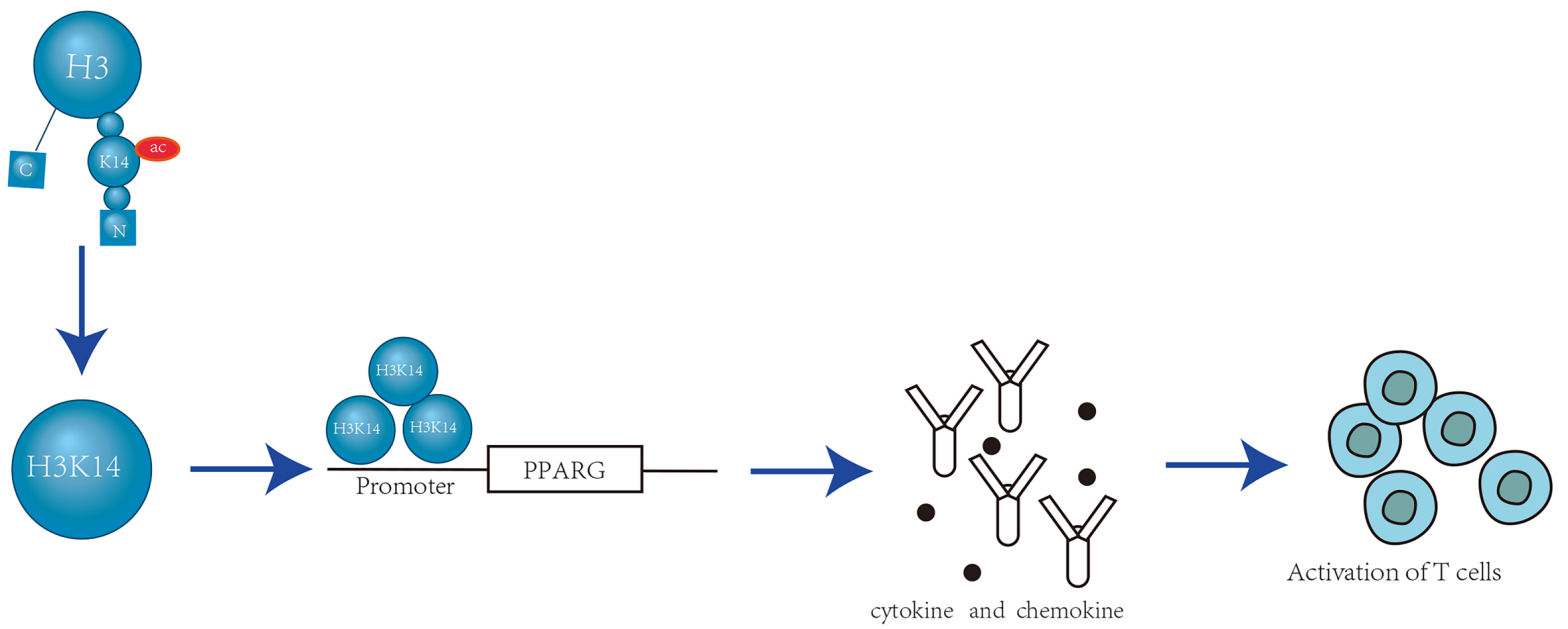
**Localization of H3K14ac at the PPARG Promoter Activates T Cells.** A local increase in histone acetylation at the PPAR-γ promoter results in enhanced transcription, which drives PPAR-γ upregulation. Elevated levels of H3 acetylation lead to its binding at the PPARG promoter. This interaction triggers the release of inflammatory chemokines and cytokines, activates T effector follicular cells, and promotes T cell activation. Consequently, it contributes to the overall inflammatory response 19.

During T cell development, the expression of CD8 is controlled by the dynamic regulation of its cis-regulatory enhancer elements. Brd1, a subunit of the histone acetyltransferase (HATs) complex, localizes to known enhancers within the CD8 gene and is responsible for acetylation at H3K14. Brd1-mediated HATs activity is crucial for effectively activating CD8 expression through H3K14 acetylation (H3K14ac). H3K14 serves as an epigenetic marker that promotes the transcription mechanism by recruiting the CD8 enhancer 18. During T cell activation, H3 (especially H3K9, H3K14 and H3K18) acetylation is localized to the promoters of IL-4, Roquin, and peroxisome proliferator-activated receptor-γ (PPAR-γ). Depletion of HDAC9 increases H3K14ac, leading to inflammation and the production of cytokines and chemokines due to overexpression of PPAR-γ 19 (Figure 2).

Follicular T helper (Tfh) cells, induced by stimulating total positive T effector cells, activate T cells, and simultaneously, increased H3K14 acetylation at the IL-4 gene promoter enhances the percentage of TH2 T cells 19, 20. IL-35, an anti-inflammatory cytokine, can suppress the immune response by inducing regulatory T cells (Tregs), regulatory B cells (Bregs), and inhibiting the effector functions of T cells and macrophages. IL-35 has been found to be induced during the development of atherosclerosis and also inhibits the activation of endothelial cells mediated by mitochondrial reactive oxygen species (mtROS) - H3K14 acetylation - AP-1 17. This suggests that inhibiting H3K14 acetylation can suppress T cell function. Maintaining a normal level of H3K14 acetylation is essential for the proper functioning of the human immune system.

### 2.4 H3K14 acetylation and propionyl, butyl combination, promote the expression of genes

H3K14 acetylation (H3K14ac) can regulate gene expression in conjunction with propionylation and butyylation. The combination of these histone modifications, including H3K14 propionylation (H3K14pr) and H3K14 butyrylation (H3K14bu), is facilitated by the deposition and enrichment of histone acetyltransferases (HATs) at active gene promoters. These modifications are recognized by specific proteins that interpret the acylation state of the histones. Moreover, propionyl-CoA can stimulate transcription in an in vitro transcription system, indicating its direct role in transcriptional regulation. Although the propionylation process is less common, it has a significant impact on the function of histone H3. Changes in metabolic states can redefine the genome-wide acylation spectrum of H3, and the absence of metabolic enzymes like propionyl-CoA carboxylase can alter the overall level of histone propionylation 21. The combination of H3K14 acetylation, propionylation, and butyrylation promotes efficient transcription and links cell metabolism with the structure and function of chromatin.

## 3. H3K14 Methylation and Ubiquitination Affect the Function of Chromatin

### 3.1 H3K14 methylation impairs DNA replication

Some methyltransferases are known to promote H3K14 methylation. For instance, Legionella pneumophila, a pathogen that causes human pneumonia, secretes an effector eukaryotic methyltransferase called RomA. RomA, which contains a SET domain, interacts with the histone H3 tail through its C-terminal anchor protein repeat sequence and trimethylates histone H3K14 through its N-terminal methyltransferase domain 22, 23. Upon localization to the infected nucleus, RomA induces a surge in H3K14 methylation, which in turn reduces H3K14 acetylation, leading to the repression of host gene expression. Additionally, certain strains of Lactobacillus and eosinophilic lung pathogens secrete a eukaryotic histone acetylation enzyme known as LphD (*Legionella pneumophila* histone Deacetylase). LphD and RomA specifically targets host chromatin. They interact with HBO1/KAT7 complexes that target H3K14, thus inhibiting its biological activity^24^. In addition to RomA and LphD, another methyltransferase containing the SET domain, SETD2, also promotes the methylation of H3K14. SETD2 mediates H3K14me3 through direct interactions with replication proteins like RPA70, which leads to the recruitment of the RPA complex to chromati^25^.

**Figure 3:**
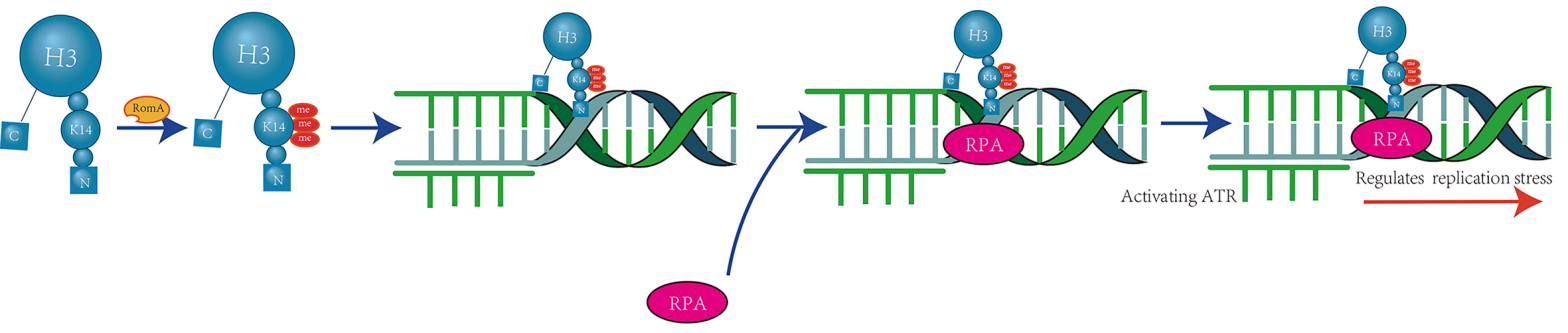
** RomA-Mediated H3K14 Trimethylation at DNA Replication Forks.** RomA, a nuclear methyltransferase, utilizes its SET domain to localize to the nucleus where it specifically recognizes and binds to the H3K14 site. It catalyzes the trimethylation of H3K14, generating the H3K14me3 mark. This modification enhances the recruitment of replication protein A (RPA) complexes to chromatin, which promotes the activation of the ATR kinase. Consequently, this regulates the cellular response to DNA replication stress under conditions that challenge the replication process 22.

H3K14 methylation is closely associated with human tumors. Studies have utilized immune slot blotting and peptide competition assays on nuclear extracts from human cervical cancer cells (HeLa) to detect H3K14 methylation. It has been observed that when cells are arrested in the S phase using double thymidine treatment, the levels of H3K14me3 (trimethylation of H3K14) increase. Isolation of proteins on nascent DNA assays revealed that H3K14me3 is enriched at stalled replication forks (Figure 3).

These findings suggest that H3K14 methylation plays a role in the DNA replication process, making cells more sensitive to replication stress. When replication forks are stalled, they cannot be efficiently restarted, leading to a prolonged cell cycle process 22. Furthermore, methylated H3K14 interacts with the epigenetic factor UHRF1, which inhibits the expression of the tumor suppressor gene TUSC3 and the acetylation of H3K14 by the histone acetyltransferase HBO1/KAT7 26. This interaction could potentially contribute to the development of tumors.

### 3.2 H3K14 ubiquitylation affects gene expression and transcriptional activity

**Figure 4:**
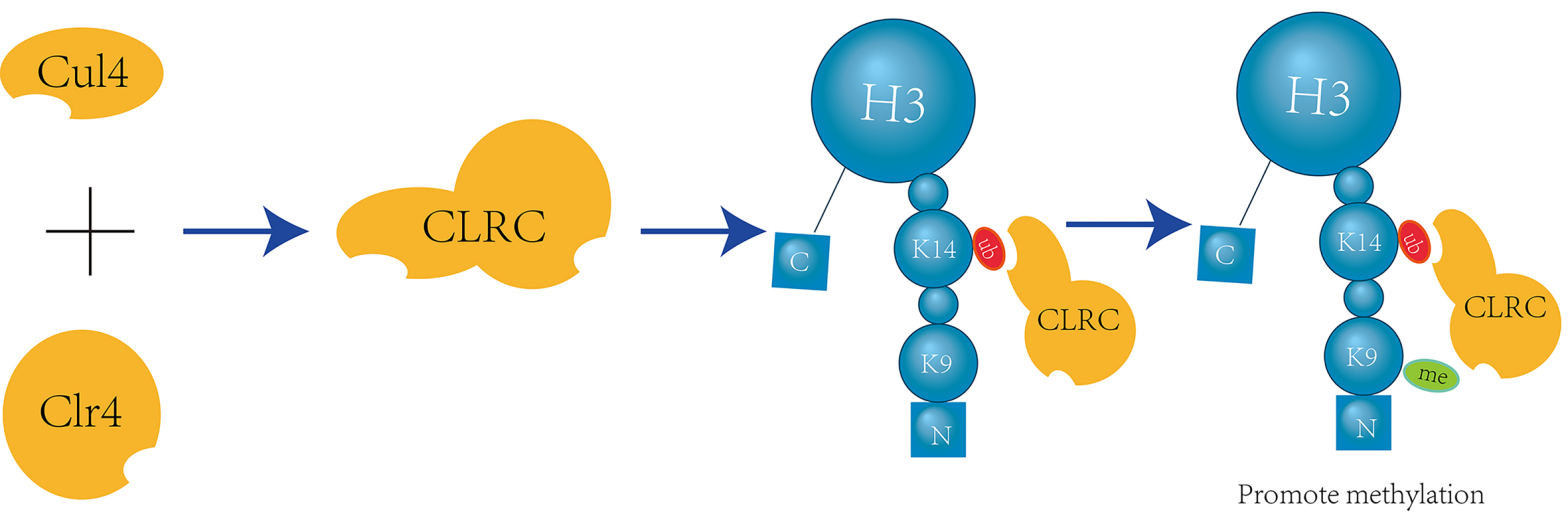
** CLRC-Mediated H3K14 Ubiquitination Facilitates H3K9 Methylation.** The E3 ubiquitin ligase Cul4 and the methyltransferase Clr4 form a multifunctional complex known as CLRC, which is involved in both ubiquitination and methylation processes. Within this complex, Cul4 specifically targets H3K14 for ubiquitination, which in turn enhances the activity of Clr4 to recognize and methylate H3K9^27^.

Crosstalk exists between H3K14 ubiquitination and H3K9 methylation, with H3K14 ubiquitination promoting H3K9 methylation in a unidirectional manner. The ubiquitin E3 ligase Cul4 and the methyltransferase Clr4 form a complex known as CLRC. Through in vitro assays and mass spectrometry (LC-MS/MS) analysis of single and double ubiquitinated H3N-GST material mediated by CLRC, it has been determined that K14 is one of the primary sites for H3 ubiquitination and can interact with the ubiquitin E3 ligase CLRC component Cul4^27^ (Figure 4).

Furthermore, using chromatin immunoprecipitation (ChIP) with different antibodies specific for H3K9 methylation, it was found that almost all trypsin-digested H3 peptides containing K14 ubiquitination also exhibit dimethylation or trimethylation of H3K9 27. This finding provides evidence for the crosstalk between H3K14 ubiquitination and H3K9 methylation.

H3K9 methylation is associated with the silencing of genes in heterochromatin 28, and an excessive level of H3K9 methylation can result in increased gene silencing. The H3K9 mutant, H3K9M, impedes the formation of major heterochromatic domains. Meanwhile, the CLRC complex catalyzes H3K14 ubiquitination in heterochromatic regions, which enhances the affinity of Clr4 for the H3 tail and boosts its enzymatic activity. This leads to elevated Clr4 levels within heterochromatin in vivo. Ultimately, the interaction between Clr4 and H3K9M prevents Clr4 from binding to H3K9, thereby blocking H3K9 methylation 29. Both an overabundance and a deficiency of H3K9 methylation can disrupt the normal dynamic balance between euchromatin and heterochromatin. Additionally, H3K14 ubiquitination can promote the progression of H3K9 methylation, potentially disrupting this delicate balance.

## 4. The Influence of Upstream Regulators of H3K14 on Its Function

### 4.1 Effect of non-coding RNA on H3K14 function

**Figure 5:**
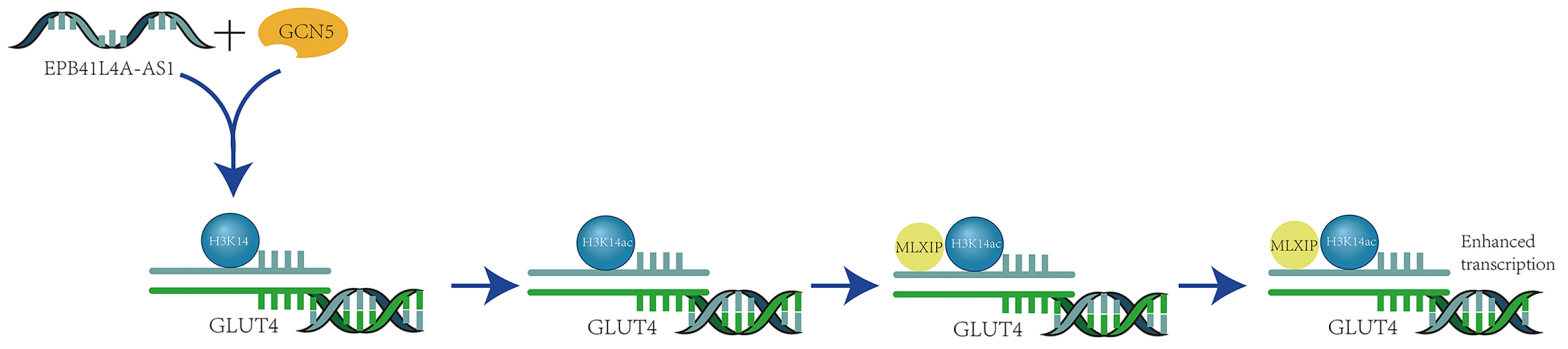
** Long Non-coding RNA EPB41L4A-AS1 Enhances H3K14 Acetylation and Transcription via GCN5 Binding.** The long non-coding RNA EPB41L4A-AS1 interacts with the histone acetyltransferase GCN5, enhancing its function and promoting the acetylation of H3K14. Once acetylated, H3K14 at the GLUT4 locus recruits the transcriptional activator MLXIP. This recruitment enhances the transcription of the GLUT4 gene and boosts GLUT4 endocytosis, thereby modulating glucose homeostasis 30.

Certain non-coding RNAs can influence the acetylation process of H3K14, thereby impacting cellular transcriptional activation. For instance, in patients with type 2 diabetes, the long non-coding RNA EPB41L4A-AS1 binds to GCN5, enhancing H3K14 acetylation in the TXNIP promoter region (Figure 5).

This interaction inhibits the thioredoxin system's function, leading to increased oxidative stress, suppressed cell proliferation, and the induction of apoptosis. Additionally, it activates transcription by promoting the recruitment of the transcription activator MLXIP, which enhances GLUT4/2 endocytosis and further inhibits glucose uptake 30. Meanwhile, the long non-coding RNA AFAP1-AS1, which is associated with actin filaments, plays a significant role in various cancers. It is upregulated in nasopharyngeal carcinoma (NPC) and serves as a poor prognostic indicator for NPC patients. AFAP1-AS1 also promotes the activation of acetyltransferase at two specific residues (E570/D610) on KAT2B. This activation further enhances H3K14 acetylation and protein binding to the bromodomain of TIF1α, which acts as a nuclear transcriptional coactivator for RBM3 transcription. This series of events leads to the stabilization of YAP mRNA, thereby enhancing the tumorigenicity of NPC 31.

In addition to long non-coding RNAs, small non-coding RNAs, such as small interfering RNAs (siRNAs), also play a role in promoting H3K14 acetylation. Studies on Spt6 mutants have revealed increased H3K14 acetylation, reduced recruitment of certain silencing factors, and impaired heterochromatin spreading when siRNAs are lost^32^. This suggests a link between siRNA function and H3K14 acetylation. At the same time, microinjection of a specific siRNA (si299) into parthenogenetic embryos increased H3K14 acetylation by targeting HDAC1 and down-regulating its functional level^33^. These findings indicate that siRNA can promote H3K14 acetylation, which in turn can inhibit tumor proliferation and the expression of certain genes.

### 4.2 Histone acetyltransferase promotes acetylation of H3K14

Histone acetyltransferases (HATs) are enzymes that acetylate histones. Based on the nature of their substrates, HATs can be categorized into two families: the GNAT family and the MYST family. Functionally, the GNAT family is primarily responsible for the acetylation of lysine residues on histone H3, while the MYST family is mainly associated with the acetylation of lysine residues on histone H4. They intersect in a few cases, such as TIP60 (MYST family) also acetylate H3K14, while PCAF (GNAT family) may be involved in H4 acetylation. In addition, they also have synergistic effects, such as GCN5 and TIP60 cooperate to activate stress response genes. The downregulation of H3K14 acetylation (H3K14ac), achieved by inhibiting histone acetyltransferases, can result in transcriptional dysregulation.

**Figure 6:**
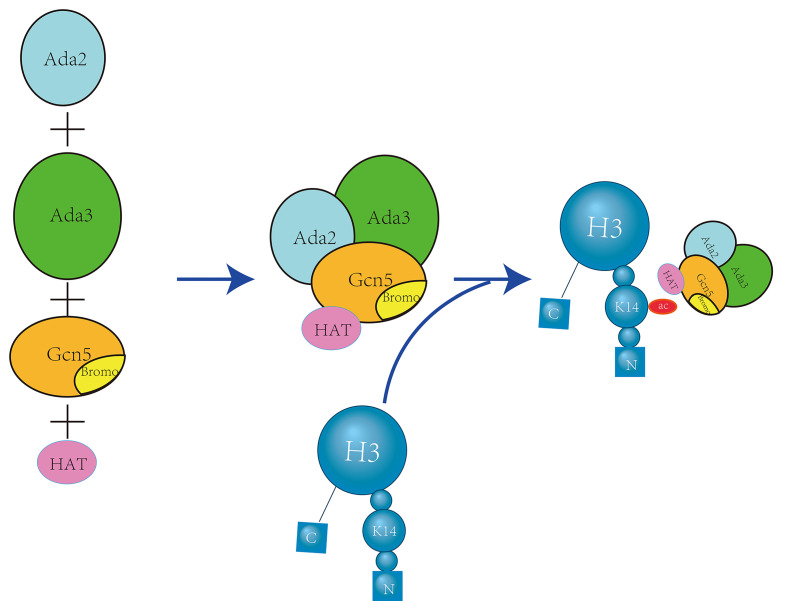
** Gcn5 Forms an ADA Complex with Ada2 and Ada3 to Specifically Acetylate H3K14.** The proteins Ada2, Ada3, and Gcn5 come together to form the ADA complex. Within this complex, the bromodomain and histone acetyltransferase (HAT) domains of Gcn5 specifically recognize and acetylate H3K14, a critical modification for transcriptional regulation 34.

GCN5 is a member of the GNAT family of histone acetyltransferases (HATs) that primarily regulates H3K14 acetylation. It contains an acetyl-lysine binding domain that is involved in the regulation of nucleosome acetylation in vitro, as well as gene promoter acetylation within cells. GCN5 binds to Ada2 and Ada3 to form the catalytic module of the ADA and SAGA transcriptional coactivator complexes (Figure 6).

Notably, the ADA subcomplex exhibits the highest acetylation specificity for H3K14^34^, which is responsible for the specific acetylation of H3K14 by GCN5. Concurrently, GCN5L can bind to the TRRAP polypeptide and is influenced by CDK8 to enhance the acetylation of H3K14 by GCN5L within the complex 35.

GCN5 regulates the expression of DKK1, an inhibitor of the Wnt/β-Catenin signaling pathway, through H3K14 acetylation, thereby influencing tumorigenesis. Beyond its direct effects, GCN5 can also interact with other proteins. A loss of GCN5 and SAS3, another GNAT family enzyme, correlates with a significant decrease in H3K14 acetylation 36. The interaction between GCN5 and CLR3 controls the acetylation level of histone H3K14 in highly expressed genes 37, helping to maintain H3K14 acetylation at specific levels. Deletion of p300/CBP-associated factor (PCAF), a histone acetyltransferase from the GCN5 family, leads to a significant reduction in the acetylation level of H3K14 residues within the chromatin of certain genes in vivo 38. Additionally, the induction of acetylated H3K14 on the p21 promoter by the tumor suppressor gene p53 is dependent on the physiological levels of PCAF 39. The STAT3 gene's expression recruits p300/CBP, promoting histone H3K14 acetylation 40. GCN5 can also regulate the global level of H3K14 acetylation in conjunction with the H3K14 acetyltransferase complex Mst2, and is capable of mediating global gene transcription and/or post-translational regulation through H3K14 acetylation 41.

HBO1/KAT7 is an enzyme belonging to the MYST family of histone acetyltransferases (HATs) that predominantly affects H3K14 acetylation. It is highly specific, and a deficiency in HBO1/KAT7 results in a 14% reduction in histone H3K14 acetylation 42. ING4 and ING5 are key subunits of the KAT7, KAT6A, and KAT6B acetyltransferase complexes. They promote the specific acetylation of H3K14ac by binding to these complexes. In Ing4^-/-^Ing5^-/-^ mouse embryonic fibroblasts (MEFs), H3K14ac levels were significantly reduced to the lower limit of detection 43. HBO1/KAT7 plays a crucial role in development as a transcriptional activator, which is essential for maintaining H3K14 acetylation (H3K14ac). In the Ser50/53Ala mutant, an alternative endogenous HBO1/KAT7 sustains histone H3K14 acetylation and responds to cell cycle regulation under UV irradiation 44. The bromodomain and PHD finger-containing protein 3 (BRPF3) forms a complex with HBO1/KAT7 and specifically acetylates histone H3K14 to facilitate DNA replication 45. BRD1 and HBO1/KAT7 are primarily co-localized in the genome, and the overall acetylation level of histone H3K14 is significantly reduced in BRD1-deficient erythroblasts 46. Meanwhile, bromodomains proteins Brd2 and Brd3 are associated with highly acetylated chromatin, and chromatin related to Brd2 and Brd3 is significantly enriched during H3K14 acetylation 47. The expression of MYST family histone acetyltransferase genes is also linked to cisplatin resistance. Studies have shown that Tip60 is overexpressed in cisplatin-resistant cells, and H3K14 is also found to be highly acetylated in these cells 48. Regardless of the diverse mechanisms of action among these acetyltransferases, MYST family histone acetyltransferases can trigger stimulus-dependent activation of inactive genes through H3K14 acetylation 49.

### 4.3 Histone deacetylases inhibit acetylation of H3K14

Histone deacetylases (HDACs) facilitate the deacetylation of histone acetyl groups, which promotes the dissociation of DNA from histone octamers. This process prevents various transcription factors and coactivators from binding specifically to DNA binding sites, thereby inhibiting gene transcription. Visualization of nucleosomes revealed that SIRT6 can gradually remove H3K14 acetylation 50. SIRT1 downregulates p53 function through deacetylation, which localizes to the promoters of several abnormally silenced tumor suppressor genes (TSGs). Only type I and II HDACs, through the deacetylation of histone H3K14, are associated with TSG silencing 51. Activation of AMPK can lead to increased gene and protein expression of SIRT1, and concurrently result in the deacetylation of H3K14^52^. Histone deacetylase inhibitors (HDACi) inhibit HDACs and directly affect the histones associated with FXN, increasing the acetylation of specific lysine residues on histone H3K14^53^. This leads to an overall increase in H3K14 acetylation.

### 4.4 Nuclear proteins promote acetylation of H3K14

The specific levels of histone H3 modifications are regulated in a variant-specific manner by members of the nucleosome-binding high mobility group N (HMGN) protein family. For instance, HMGN2 can significantly enhance H3K14 acetylation 54. Concurrently, the binding of HMGN3a/b results in increased acetylation of histone H3K14, which in turn stimulates Glyt1a expression. This stimulation can lead to more efficient transcription elongation and increased mRNA production 55. Depletion of HMGN1 reduces the level of Hsp70 transcripts early in heat shock. It also increases H3K14 acetylation in Hsp70 chromatin more efficiently in wild-type cells, thus accelerating the rate of chromatin remodeling during the early phase of Hsp70 activation 56. These findings demonstrate the role of nucleosome proteins in regulating H3K14 acetylation.

Furthermore, it has been observed that the loss of function of the nuclear protein Sde2 results in an increased occupancy level of acetylated histone H3K14 and RNA polymerase II at telomeres. This also leads to a decreased recruitment of SHREC to telomeres, thereby impairing the transcriptional silencing of these regions 57. Similarly, the overexpression of another conserved nuclear protein, Epe1, which encodes a JmjC domain, has an analogous effect. It disrupts heterochromatin formation by increasing the acetylation of H3K14^58^.

### 4.5 Influence of other factors on H3K14 function

In addition to the epigenetic factors previously discussed, other elements such as pharmaceutical compounds and daily dietary habits also influence H3K14 modification. The interplay of these diverse factors may impact the expression levels of enzymes involved in the acetylation and deacetylation processes of H3K14.

Numerous exogenous drug compounds are known to influence H3K14 modification. Upon examination of the data presented in Table 1, it becomes evident that the mechanisms by which some of these drugs affect H3K14 modification remain unclear and require further investigation. Nevertheless, it has been established that H3K14 modification can impact the activity of histone acetyltransferases (HATs) or histone deacetylases (HDACs), offering a potential avenue for the development of treatments for related diseases (Table 1).

In addition to pharmaceutical drugs, an individual's lifestyle choices and living environment can significantly impact H3K14 modification. Moderate alcohol consumption, for instance, has been shown to promote H3K14 acetylation. Experiments with acute ethanol exposure have demonstrated that alcohol can reduce HDAC activity and the HDAC2 protein level in the amygdala, thereby increasing overall histone acetylation, including specificity for genes such as BDNF and Arc 59. Caffeine may have the opposite effect, potentially reducing H3K14 acetylation. Prenatal caffeine exposure (PCE) has been found to inhibit insulin-like growth factor 1 (IGF1) and to significantly increase the mRNA expression of DNA methyltransferases (DNMT1 and DNMT3A) as well as HDACs (HDAC1 and HDAC2), which in turn reduces H3K14 acetylation levels^60^. Furthermore, a high sugar diet can inhibit the expression of SIRT2, leading to accelerated H3K14 acetylation, increased ROS levels, and the activation of inflammatory responses^61^. Diet is not the only factor; behaviors such as smoking also affect H3K14 modification. Smoking significantly reduces the expression of the HBO1/KAT7 protein and the level of H3K14 acetylation^62^. Additionally, components like nicotine and lipopolysaccharides in cigarette smoke have been shown to decrease H3K14 acetylation^63^, ^64^. Special environmental conditions can also alter H3K14 modification. Exposure to PM2.5 and cold stress (PMCS) can lead to increased inflammation and REDOX levels. This exposure also increases the percentage of TH2 T cells by regulating P300 and HDAC1, thereby increasing H3K14 acetylation^20^, and can induce the progression of immune responses.

In addition to exogenous influences, the body's own internal changes or mechanisms, such as DNA repair and gene expression mutations, can also affect H3K14 modifications. Mutations in Mismatch Repair (MMR) can cause a redistribution of the Sir2 deacetylase, leading to higher acetylation levels of H3K14 at silent mating sites and telomeres, and lower acetylation levels in rDNA^65^. Mutations in the thyroid hormone receptor (TR), specifically the F455S mutation, can result in H3K14 acetylation even in the absence of thyroid hormone^66^. Interferon regulatory factors (IRF) 3 and IRF7 can enhance H3K14 acetylation, while the association of HDAC3 with TBP can inhibit H3K14 acetylation. The activation of IRF7 not only boosts H3K14 acetylation but also increases the expression of the IFN-A gene 67.

A variety of domains and proteins significantly influence H3K14 modification. The YEATS domain is a novel acetyl-lysine binding module, with GAS41 being a key component of chromatin remodeling 68. GAS41 colocalizes with H3K14ac on the promoters of actively transcribed genes. The absence of GAS41 or its YEATS domain, or the disruption of interactions with acetylated histones, can inhibit the growth and survival of cancer cells both in vitro and in vivo^69^. The KRAB domain-associated protein 1 (KAP1) acts as a cofactor that inhibits the transcription of target genes by reducing H3K14 acetylation^70^. In yeast, the conserved histone acetyltransferase complex NuA3, through its Yng1 subunit (PHD finger), specifically binds to H3K4me3 and then catalyzes H3K14 acetylation via the Sas3 histone acetyltransferase domain, initiating transcription of a subset of genes^71^. MORF mononuclear cell leukemia zinc finger protein and related factors are associated with tumor formation. Its unique structure domain, DPF, recognizes binding sites on H3K14. DPF, in collaboration with the MYST domain, promotes H3K14 acetylation^72^. Additionally, BRPF1 can enhance H3K14 acetylation at promoters through the MOZ/MORF complex^46^. These proteins are not directly histone modification enzymes, but their structural domains affect H3K14 modification, suggesting they could serve as targets for the treatment of related diseases.

## 5. The Relationship Between the Modification of H3K14 and the Diagnosis, Occurrence, Treatment of Diseases

In general, H3K14-related diseases can be detected by assessing the degree of H3K14 modification or by evaluating the concentrations of histone acetyltransferases (HATs) and histone deacetylases (HDACs). However, these factors are not always easy to measure directly. A compound of interest is galectin-9, the levels of which can deviate from normal values in certain cancers. The transcriptional regulation of the LGALS9 gene, which encodes galectin-9, is influenced by histone acetylation^94^. As a result, galectin-9 levels have emerged as a novel biomarker for detecting diseases associated with H3K14 modifications.

The relationship between different diseases and H3K14 varies, as does the nature of existing therapeutic targets. As illustrated in Table 2, it is evident that the association between H3K14-related diseases and the level of H3K14 acetylation, as well as the interplay between H3K14 acetylation and DNA methylation, differ significantly (Table 2).

From the table above, we have gained a certain understanding of the potential therapeutic targets for various diseases. These targets are generally approached by modulating DNA methylation and the activity of histone acetyltransferases (HATs) and histone deacetylases (HDACs) that regulate H3K14 acetylation. HDAC inhibitors (HDACi) have proven to be effective anticancer drugs, serving as candidates to combat invasive malignant tumors 58. Additionally, therapies may be directed at specific signaling pathways or gene expressions. In the latest research, an additional method has emerged as a potential treatment for H3K14-related diseases: the timely response to double-strand breaks in chromosomal DNA (DSBs).

An effective and accurate response to double-strand breaks (DSBs) is crucial for maintaining genomic stability and preventing chromosomal changes that can lead to cancer. The production of DSBs is associated with structural changes in chromatin and the activation of the protein kinase ataxia-telangiectasia mutated (ATM). ATM is a key factor in the cellular response network that controls the signaling in response to DSBs. The high mobility group nucleosomal binding protein 1 (HMGN1) plays a role in modulating the interaction between ATM and chromatin before and after the formation of DSBs, thereby optimizing ATM activation. Ionizing radiation (IR) processing leads to an overall increase in H3K14 acetylation that is dependent on HMGN1. Cells treated with histone acetylation enzyme inhibitors around HMGN1 can effectively activate ATM and timely respond to DSBs 95. Additionally, DNA-dependent protein kinase (DNA-PK) is involved in DSB signal transduction and repair. Histone acetylation enzyme inhibitors, such as trichostatin A, can lead to the phosphorylation and repositioning of DNA-PK to DNA. An increase in H3K14 acetylation occurs in hypoxic cells. The activation of DNA-PK by hypoxia positively regulates the key transcription factor HIF-1 and its subsequent target gene GLUT1, thereby actively modulating cellular oxygen sensing and signaling pathways 96.

## 6. Summary and Outlook

Current research on H3K14 focuses more on the role of histone modifications in diseases, particularly the acetylation process of H3K14, which plays a significant role in gene expression and the initiation of the immune system. While most research concentrates on H3K14 acetylation, studies on H3K14 methylation and other modifications are not as in-depth. When exploring diseases, it's important to consider not only acetylation but also other factors and perspectives.

H3K14 modifications are influenced by many drug compounds, primarily affecting the upstream regulators, namely, the expression of histone acetyltransferases (HATs) and histone deacetylases (HDACs), which regulate the H3K14 acetylation process. Research on this subject and associated diseases helps to clarify the mechanisms of disease more clearly.

Detecting galectin-9 levels as a method to identify diseases associated with H3K14 is relatively straightforward. According to common research perspectives, using HDAC inhibitors (HDACi) to treat H3K14-related diseases may be one of the most commonly employed measures. In summary, current research has enhanced our understanding of the mechanisms and therapeutic principles of H3K14. However, most studies on H3K14 modifications focus on acetylation, with less emphasis on other modification mechanisms. The focus on H3K14-related diseases still largely relies on the use of HDAC inhibitors, with few alternative therapeutic approaches. Therefore, it is essential to continue studying and identifying potential therapeutic targets for the treatment of H3K14-related diseases.

## Figures and Tables

**Table 1:** Mechanisms and Effects of pharmacological compounds on H3K14 modification

Name of Drug Compound	Mechanism of action	Results	References
Ochratoxin A (OTA)	Mediates downregulation of acetylation genes	Loss of acetylation at H3K14	73
Hydroxamic acid derivative CTS203	Inhibition of HDAC activity	H3K14ac goes up	74
Isoniazid (INH)	Increased expression of HDAC1 and HDAC2	H3K14ac decreases and SOD activity decreases	75
Scriptaid	Scriptaid inhibits the expression of the HDAC5 gene	H3K14ac goes up	76
Butyrate	Inhibition of HDAC	H3K14ac goes up	77
Valproate (VPA)	Activation of BDNF-TrkB signaling pathway and inhibition of HDAC	H3K14ac goes up	78, 79
Compound 511	The mRNA expression of HDAC1/6/8 was up-regulated	Inhibits morphine-induced H3K14 hyperacetylation	80
Bisphenol A (BPA)	Enhanced HDAC2 expression	Reduces histone acetylation of the StAR promoter	81
LPC	Up-regulated KAT2A, KAT5, and KAT9	H3K14ac goes up	16
Melatonin	Activation of CREB-binding protein (CBP) /p300 histone acetyltransferase activity via ERK signaling pathway	H3K14ac goes up	82
Osthole	Upregulation of MOZ and MORF	H3K14ac goes up	83
Conjugated bile acids (CBAs)	Hdacs inhibited by pernuclear s1p; And affect the CCAS-S1PR2-nuclear SphK2-S1KP signaling pathway	H3K14ac goes up	84
Green tea catechin epigallocatechin-3-gallat (EGCG)	The expression of DNMT1, HDAC1, HDAC2 and G9a was down-regulated	Enhanced and acetylated H3K14 binding to p27, PCAF, C/EBPα and C/EBPɛ promoter regions	85
Pomegranate seed Oil (PSO)	Increase IGF-1 levels	Acetylated H3K14 was enriched at the IGF-1 gene promoter	86
20(S)-ginsenoside Rh2 (Rh2)	Inhibition of total HDAC activity and induction of MAPK signaling	H3K14ac goes up	87
Black Mulberry Extract (BME)	Inhibition of total HATs and p300 activities in vitro	H3K14ac goes down	88
Paeonol	Weak h2o2 -induced upregulation of lysine deacetylase activity of Sirt1	H3K14ac goes down	89
Blueberry treatment		Regulates histone acetylation	90
Chp2 protein	Binds to the SHREC histone deacetylase complex (SHREC2)	H3K14ac goes down	91
Protein Fbxw15	Mediates HBO1/KAT7 ubiquitination and degradation	It regulates acetylation of histone H3K14	92
Dmp53	Required for the maintenance of basal H3-K14 acetylation levels in Drosophila chromatin	Maintain H3K14 acetylation levels	93

**Table 2:** Occurrence and Potential treatment of H3K14-related diseases

The name of the disease	Mechanism of impact	Potential therapeutic targets/measures	Reference
Cutaneous T Cell Lymphoma (CTCL)	Genome-wide H3K9/14ac (H3ac) and H3K27ac levels were significantly increased	HDACi	97
Renal Cell Carcinoma (RCC)	Renal oxidative stress, high DNA methylation, low H3 acetylation	Raise the level of oxidative stress	98
Acute promyelocytic leukemia (APL)	The PML-RARA fusion protein recruits HDAC/HMT to form an inhibitory complex that blocks differentiation gene expression. Belinostat and 3-Deazaneplanocin A directly disrupt the complex and reverse the apparent silencing by H3K14ac elevation	HDAC inhibitor combined with HMT inhibitor; EGCG	85, 99
Breast cancer	Increased expression of ATP-binding cassette (ABC) -type transporters	RNAi down-regulated the expression of HATs PCAF and GCN5	100
Abdominal Aortic Aneurysm (AAA)	There is increased acetylation of H3K14 and DNA hypomethylation	mRNA levels of DNMT and HDAC	101, 102
Glioma	IDH1 mutations can lead to a wide range of DNA and histone methylation; Overexpression of HCMV IE86 protein	The joint HDACi cAMP agonist, ATF5expression	10, 103, 104
Nasopharyngeal carcinoma	Upregulation of AFAP1-AS1, H3K14acetylation and protein binding to the brominated domain of TIF1α	Enhanced KAT2Bacetyltransferase activation and YAP mRNA stabilization	31
Periodontitis	Cut GCN5 activated PDLSCs Wnt/beta Catenin signaling pathway, H3K14ac level is low	Up-regulation of GCN5	83, 105
Mastitis	H3K14 high acetylation, lipopolysaccharide	Metformin activates AMPK signaling pathway to reverse H3K14hyperacetylation	106-108
Acute necrotizing pancreatitis (AP)	The key initiating cytokine TNF-AP was strongly up-regulated and H3K14 acetylation was increased		109
Asthma	PMCS, increasethe CD4 + T cells inthe IL - 4 gene promoter H3K14 acetylation and H3K27me3	Notch Signaling pathway	20, 110
Diabetes	SIRT2 expression was decreased, EPB41L4A-AS1 binding to GCN5, andH3K14 level was increased. Claudin-5 is deficient	Upregulation of SIRT2; EPB41L4A-AS1; Ketoβ-hydroxybutyricacid (BHB) therapy	30, 61, 111, 112
Neurodegenerative disease Friedreich's ataxia	FXN introns within the GAA x TTC triplets amplification result in transcriptional silencing, with histone H3 acetylation	HDACi	53
Intrauterine growth retardation (IUGR)	Prenatal caffeine exposure increased the mRNA expression of DNAmethyltransferases Dnmt1, Dnmt3a, HDAC1, and HDAC2, and decreased the acetylation level of H3K14	DNA methylation and histone acetylation	113, 114
Tuberculosis (TB)	HDAC1 gene/protein expression increases, H3K14Ac decrease	Downregulation of HDAC	115
